# Mutual Inductance and Coupling Effects in Acoustic Resonant Unit Cells

**DOI:** 10.3390/ma12091558

**Published:** 2019-05-13

**Authors:** Changlin Ding, Yibao Dong, Kun Song, Shilong Zhai, Yuanbo Wang, Xiaopeng Zhao

**Affiliations:** Department of Applied Physics, Northwestern Polytechnical University, Xi’an 710129, China; 2012302768@mail.nwpu.edu.cn (Y.D.); shilongzhai@nwpu.edu.cn (S.Z.); yuanbowang@mail.nwpu.edu.cn (Y.W.)

**Keywords:** mutual inductance, dumbbell-shaped split hollow sphere (DSSHS), acoustic metamaterial (AMM), acoustic metasurface (AMS), broadband

## Abstract

We present an acoustic metamaterial (AMM) consisting of a dumbbell-shaped split hollow sphere (DSSHS). Transmission results of experiments and simulations both presented a transmitted dip at the resonant frequency of AMM, which demonstrated its negative modulus property. As the two split holes in the DSSHS had strong coupling effects for the acoustic medium in the local region, the dip could be simply manipulated by tuning the distance between the split holes. When the distance was large enough, the mutual inductance tended to disappear, and a weak interaction existed in the structure. According to the property of weak interaction, a multiband AMM and a broadband AMM with a negative modulus could be achieved by arraying DSSHS clusters with different distances. Furthermore, mutual inductance and coupling in DSSHS reinforced the local resonance, and this kind of cell could be used to design the acoustic metasurface to abnormally control the refractive waves.

## 1. Introduction

The proposal of metamaterials [1,2] accelerates the development of abnormal manipulations of waves. Electromagnetic (EM) metamaterials, or metasurfaces, are artificial materials fabricated by top-down or bottom-up approaches [3,4]. They have manipulated EM waves in unusual ways such as negative refraction, sub-wavelength imaging, cloaking, absorption, trapped rainbow effect, polarization conversion, optical vortex, and others [5,6,7,8,9,10,11]. As the concept of local resonance is introduced into the acoustic field [12], acoustic metamaterials (AMMs) (bulk material) or metasurfaces [13] (with subwavelength thickness) present similar properties for acoustic waves as the EM counterparts such as double negative parameters, flat focusing, subwavelength imaging, cloaking, abnormal reflection and refraction, phase engineering, total sound absorption, topology inspired characteristics [14,15,16,17,18,19,20,21,22,23,24,25,26,27,28,29], damping ratio enhancement, the band gaps of vibration, and so on [30,31,32,33].

However, as the unusual effects of AMMs are based on the mechanism of local resonance, the bandwidth of the working frequency is very narrow. This defect of AMM hampers its application in our daily life. A Helmholtz resonator can be used to achieve AMMs with negative modulus near the resonant frequency in water medium [15]. Sheng et al. [14,16] presented a membrane-type AMM that had negative mass density or double negative parameters in a narrow band because of local resonance. From our earlier works, the response of negative parameters only occurred in a narrow band near the resonant frequency [18,20,34,35]. Therefore, design of the broadband AMMs is very important. One path is to combine the different sized resonant cells in a group to achieve multiband and/or broadband AMMs, in which each cell exerts its function and oscillates in its local resonant frequency band, and the superposition of the local resonance will produce broadband effects [19,36,37,38,39]. By coupling split hollow spheres (SHSs) with different geometry sizes, a multiband and broadband AMM with negative moduli can be realized [19]. Yang et al. used membranes with different weights to achieve broadband sound attenuation [36]. Another method is coupling local resonance with Bragg band gaps [40,41]. Bragg band gaps in phononic crystals are based on the mechanism of Bragg scattering, in which the working wavelengths are comparable with the lattice constants [42,43,44]. Local resonance band gaps are based on resonant structures with subwavelength sizes less than λ/3. Furthermore, some other kinds of passively and actively tunable AMMs have been presented to study the broadband AMMs [45,46,47,48]. All the methods to realize broadband AMMs are based on the intrinsic properties of the unit cells. 

In this paper, we will introduce another method of resonant coupling between the unit cells to achieve a broadband AMM with a dumbbell-shaped split hollow sphere (DSSHS) structure. Resonant coupling is caused by the mutual inductance of two resonators, SHSs, which will generate abundant properties for incident acoustic waves. By tuning the distance between the two resonators, we can achieve different mutual inductances in the structure, which results in different local resonant frequencies to design broadband AMMs. The DSSHS with mutual inductance may strengthen the resonance of the SHS unit and can be applied to design a transmitted AMS.

## 2. Model Analysis

**Figure 1:**
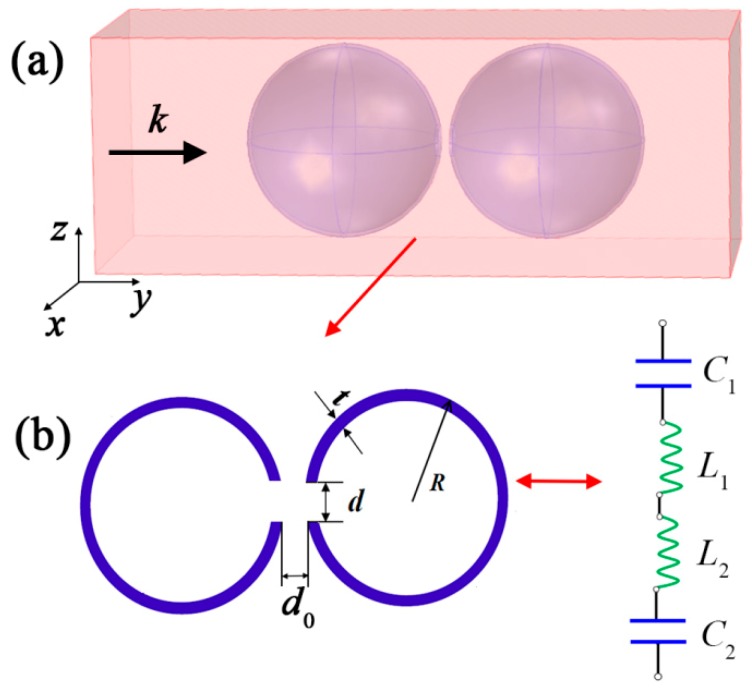
(**a**) The 3D schematic diagraph of DSSHS in acoustic waveguide; (**b**) the cross-sectional diagram of DSSHS and the acoustic effective L-C circuit.

SHS is a kind of hollow sphere with a cylinder split hole, as shown in Figure 1a. Figure 1a also gives the 3D schematic diagraph of the DSSHS model in an acoustic waveguide environment. The DSSHS consisted of two equally sized SHSs with the split holes facing each other. From the cross-sectional diagram of DSSHS in Figure 1b, the thickness of the hollow sphere was *t* = 1 mm, the radius of the hollow sphere was *R* = 25 mm, the diameter of split holes was *d* = 4 mm, and the distance between the two split holes *d*_0_ could be tuned from 0.5 to 20 mm. As presented in previous works [18,19,22,23,24], the single SHS could be considered as acoustic Helmholtz resonator (HR), and was equivalent to an acoustic L-C circuit. The resonant frequency could only be tuned passively by changing the diameter of the split holes or the cavity of hollow sphere, which should change the internal structure of SHS. However, in this work, we manipulated the distance of the split holes between the two SHSs to tune the resonant frequency, which was used to achieve active, tunable acoustic metamaterials. That is to say, the DSSHS was a modified HR and had more abundant properties. The two cavities of the DSSHS could store more energy and caused the sound medium to oscillate in and out of the two holes. The sound medium in the two SHSs coupled with each other and affected its intrinsic properties, which relied on the distance of the two split holes. Strong local coupling will occur near the two split holes. The DSSHS resonator was analogous to a capacitor–inductor–inductor–capacitor circuit. The two cavities of the DSSHS acted as two series capacitors with capacitance:

(1) $$C_{1}=C_{2}=V/(\rho c_{0}^{2}).$$

The effective acoustic capacitance is:

(2) $$C_{\mathrm{eff}}=\frac{C_{1}C_{2}}{C_{1}+C_{2}}=V/(2\rho c_{0}^{2}),$$

where *V* is the volume of the hollow cavity, and $\rho_{0}$ and $c_{0}$ are the mass density and sound velocity of the sound medium.

The two split holes were considered as two series inductors, which included the contributions of the two opening holes and the coupled mutual interaction. The effective acoustic inductance is:

(3) $$L_{\mathrm{eff}}=L_{1}+L_{2}.$$

To retrieve the relationship between the effective inductor *L*_eff_ and the distance of the two split holes *d*_0_ in the DSSHS, we introduced the acoustic inductive susceptance:

(4) $$G=\frac{1}{L_{\mathrm{eff}}}.$$

When there was no coupling effect, the inductive susceptance of the DSSHS was

(5) $$G_{0}=\frac{1}{2L_{s}}=\frac{S}{2\rho d_{s}},$$

where $L_{s}$ is the acoustic inductor of single SHS [18] structure:

(6) $$L_{s}=\rho d_{s}/S,$$

in which *S* is the cross area of the hole and *d*_s_ is the effective length of the hole related to *t* and *d*:

(7) $$S=\pi {\left(d/2\right)}^{2};$$

(8) $$d_{s}=t+1.8\sqrt{d},$$

Considering the coupling effect of the two split holes, the change rate of G was proportional to $G-G_{0}$. The relation can be expressed as:

(9) $$\frac{dG}{d(d_{0})}=k(G-G_{0}).$$

Taking the initial condition (*d*_0_ = 0, *G* = 0) into the differential Equation (9), we can obtain the solution as:

(10) $$G=G_{0}(1-e^{-k(d_{0}+\delta )}),$$

where δ is a correction factor related to the coupling effect, and *k* is a proportionality constant. According to the L-C model, the resonant frequency is:

(11) $$f=\frac{1}{2\pi \sqrt{C_{\mathrm{eff}}L_{\mathrm{eff}}}}=f_{0}\sqrt{1-e^{-k(d_{0}+\delta )}},$$

where $f_{0}$ is the resonant frequency of the single SHS. The distance of split holes *d*_0_ may tune the resonant frequency of DSSHS in an exponential way because of mutual inductance and the coupling effect. 

## 3. Results and Discussion 

### 3.1. Tunable Dumbbell-Shaped Split Hollow Sphere (DSSHS) Acoustic Metamaterial (AMM) with Negative Modulus

**Figure 2:**
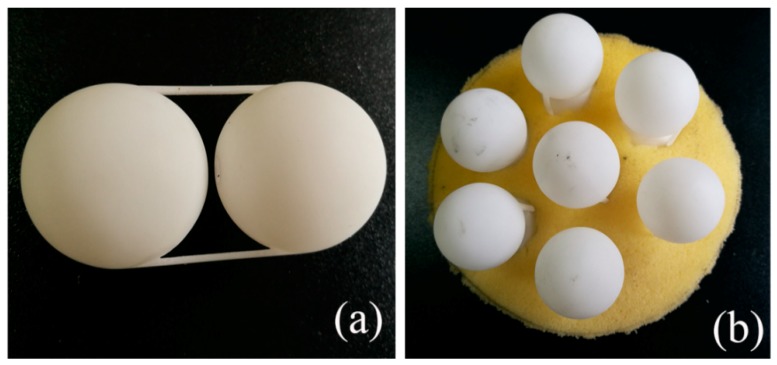
(**a**) The 3D printed DSSHS structure; (**b**) the fabricated specimen of DSSHS AMM.

Firstly, we conducted a full-wave simulation to investigate the DSSHS AMM with COMSOL Multiphysics 5.2a software (COMSOL Inc., Stockholm, Sweden) based on the finite element method (FEM). Figure 1a shows the simulation environment. The DSSHS was located in an acoustic waveguide tube with a periodic boundary, which meant that the DSSHS AMM was designed by the periodic DSSHS array. The left- and right-side faces were set as radiation boundaries. A plane-harmonic acoustic wave (1 Pa) was perpendicularly incident on the left-side face. The entire domain of the waveguide was meshed by free tetrahedrons based on the face meshing, and the user predefined size was set as ‘refine’, implying that the maximum element size was 9.6 mm, the minimum element size was 1.2 mm, and the maximum element growth rate was 1.45 mm. The model was calculated in frequency domain, and the solvers were chosen as ’MUMPS’. From the simulation, the DSSHS AMM presented a transmission dip, as shown in Figure 2a, which was similar with the single SHS AMMs. The dip could be tuned by the distance of the split holes. Notably, the transmission dip was attributed to the local resonance of the single SHS in DSSHS; however, the tuning of the dip might have been caused by the mutual coupling effect between the two SHSs.

**Figure 3:**
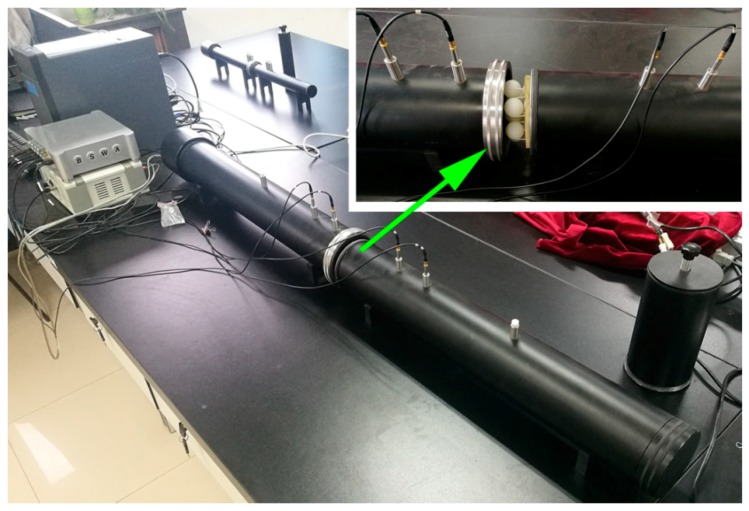
Physical diagram of the impendence tube testing equipment.

To verify the simulation, we conducted a transmission experiment of DSSHS AMM in an impedance tube system to investigate its acoustic properties. The DSSHS was fabricated by 3D printing technology using thermoplastics (Stratasys Dimension Elite, 0.1 mm in precision, Shenzhen, China), and the unit cell was fixed by two brackets composed of plastic bars, as shown in Figure 2a. The sample was prepared by embedding seven DSSHSs in the sponge matrix with a lattice constant of 30 mm, as shown in Figure 2b. Measurements of the transmission amplitude were conducted in a Shengwang transmission loss impedance tube apparatus, which was introduced in Reference [19]. The testing equipment was shown in Figure 3, which consisted of an impedance tube, four microphones, a power amplifier, a data collecting analyzer, and a computer. The diameter of the impedance tube was 100 mm, and the diameter of the DSSHS AMM sample was 100 mm. The inset in the upper right corner showed the testing sample in the impedance tube.

**Figure 4:**
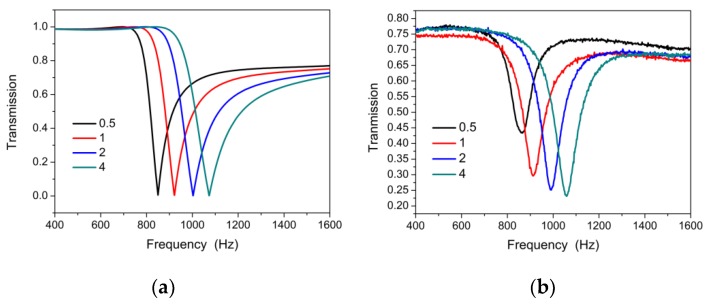
(**a**) The simulated and (**b**) experimental transmissions of the DSSHS AMMs with tuned distances, *d*_0_, from 0.5 to 4 mm.

Testing results are shown in Figure 4b. It was demonstrated that there was a transmission dip for the DSSHS AMM, and the dip could be moved by tuning the distance *d*_0_ of the structure. Experimental results agreed well with the simulation ones. It was also shown that the experimental transmission dip of the sample became deeper with an increase in the SHS’ diameter. However, the simulated transmission dip was near zero for each DSSHS AMM, which did not clearly show the change in Figure 4a. As the simulated environment was set to ideal conditions without any damping, the resonance of DSSHS resulted in the near zero transmission dip. But in experiments, the damping forces of the four DSSHS AMMs led to the different transmission dip depths, as shown in Figure 4b. 

**Figure 5:**
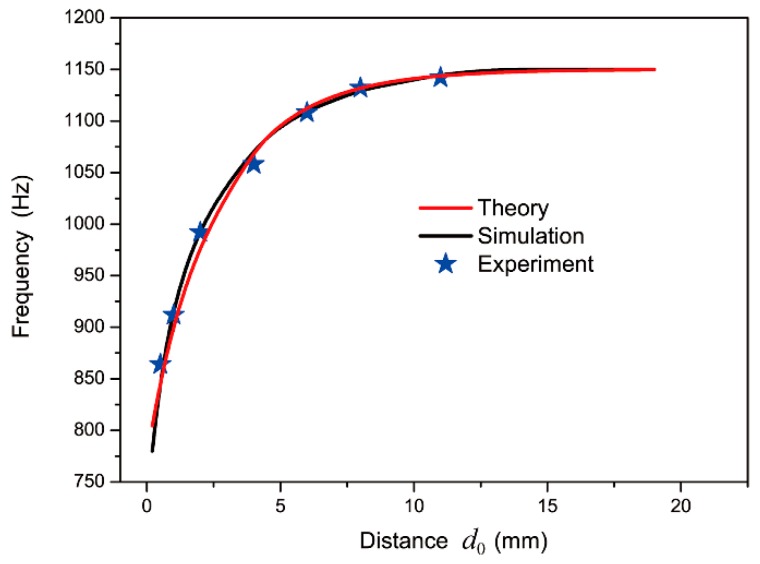
The relationship between resonant frequency and distance, *d*_0_, of two spit holes in DSSHS.

We systematically investigated the relation between resonant frequency and distance of the two split holes in DSSHS to further illustrate the tuning feature of the resonant frequency, as shown in Figure 5. The results from theoretical formulation (red curve) and the simulation based on the finite element method (black curve) agreed well with experimental data. It was clear that the resonant frequency generated blue shift as the distance *d*_0_ increased in the DSSHSs. However, when the distance *d*_0_ was larger than 8 mm, the resonant frequency was stable at 1150 Hz, which was just the local resonant frequency of the single SHS. It illustrated that there was only weak interaction between the two SHSs, without coupling effects, when the distance was large enough. When the split holes of SHSs were close to each other, the resonances in the cells affected each other and resulted in the mutual coupling effect, which led to the tuning of the resonant frequency.

**Figure 6:**
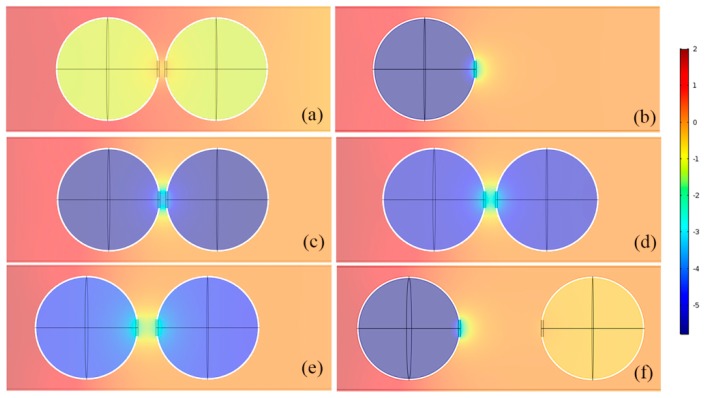
The acoustic field distribution at 1150 Hz of (**a**) DSSHS and (**b**) SHS. The transmitted acoustic field of DSSHS with different hole distances at resonant frequency, (**c**) 1, (**d**) 2, (**e**) 4, and (**f**) 19 mm.

To clearly demonstrate the physical mechanism of resonant coupling, we conducted the Comsol simulation based on the finite element method (mentioned above) to study the acoustic field distributions of the DSSHS and single SHS at each resonant frequency. It was shown in Figure 6b that the single SHS stored a large amount of acoustic energy in the cavity and released it from the split hole at 1150 Hz, which demonstrated the resonant phenomenon. That is to say, acoustic energy was stored in the cavity and released from the split hole of SHS. When the energy in two acoustic structures generated a synergistic response, the unit cell resonated to store maximum energy in the cavity and released a lot of energy out of the split hole over a wide region.

However, from Figure 6a, the internal field of the DSSHS with a hole distance of 1 mm was weak, at 1150 Hz, and the acoustic energy was close to the background pressure, which showed no resonant response. In the case of the frequency at 910 Hz, the acoustic pressure in the volume of DSSHS reached a maximum negative value, as shown in Figure 6c, which indicated resonance with a negative response. The acoustic field changed sharply near the split holes with strong mutual inductance. In this case, the acoustic media released from the split holes of the two SHSs could not oscillate freely; they entered into another SHS split hole and cavity to couple with each other, which enhanced each SHS’ effective acoustic inductance and produced a new resonant effect in a lower frequency region. Also, the acoustic oscillating energy near the split holes in DSSHS was larger than that in SHS, which stated that the mutual coupling reinforced the resonance for acoustic waves. This kind of stronger resonant structure could be applied to design transmitted AMSs, which are discussed in the next section.

Figure 6c–f shows resonant acoustic fields of four kinds of SSHSs with hole distances of 1, 2, 4, and 19 mm, respectively, which demonstrated mutual inductance in the DSSHS. Compared with the four kinds of acoustic field distributions, the acoustic energy in the cavity weakened with the increase in hole distance *d*_0_ and the mutual coupling between the two split holes. That is to say, each effective acoustic inductance became larger and larger to result in the blue shift of the resonant frequency. When the distance was large enough, as shown in Figure 6f, it was very hard to see the mutual inductance and coupling effect between two split holes in DSSHS at resonant frequency. The first cell of DSSHS presented strong resonant effects, with the same pressure distribution as the single SHS as shown in Figure 6b, while the energy in the second cell was weak and close to the background pressure, which indicated weak interaction between two resonant cells in DSSHS. To sum up, the different distances between the two split holes resulted in changeable coupling effects, which could be used to control the resonant frequency of DSSHS. According to weak interaction, we designed a kind of broadband AMM.

### 3.2. Broadband DSSHS AMM

**Figure 7:**
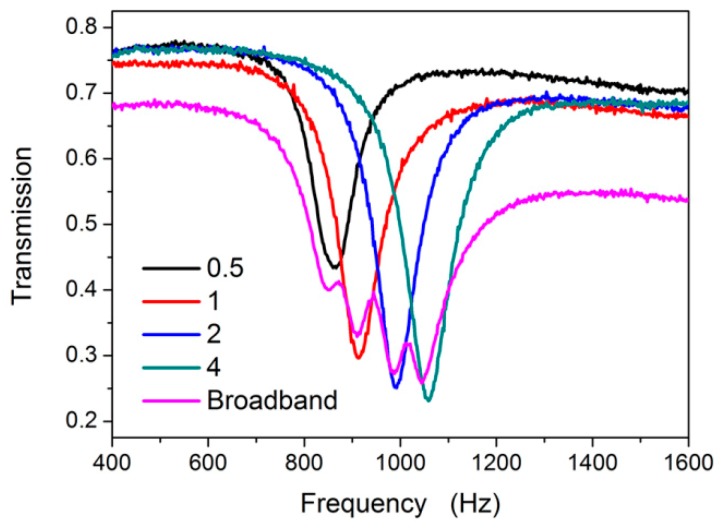
The transmission of broadband AMM.

The broadband sample was fabricated by filling four kinds of DSSHSs with distances of 0.5, 1, 2, and 4 mm. The experimental transmission results are shown in Figure 7. There were four transmission dips in accordance with the each DSSHS sample, which illustrated weak interactions among the different DSSHSs. Because the resonant frequencies were very close to each other, the resonant districts overlapped and became a broadband transmission dip. From the half maximum peak of the transmission curve, it was demonstrated that the broadband dip was located in the frequency range of 820~1110 Hz.

**Figure 8:**
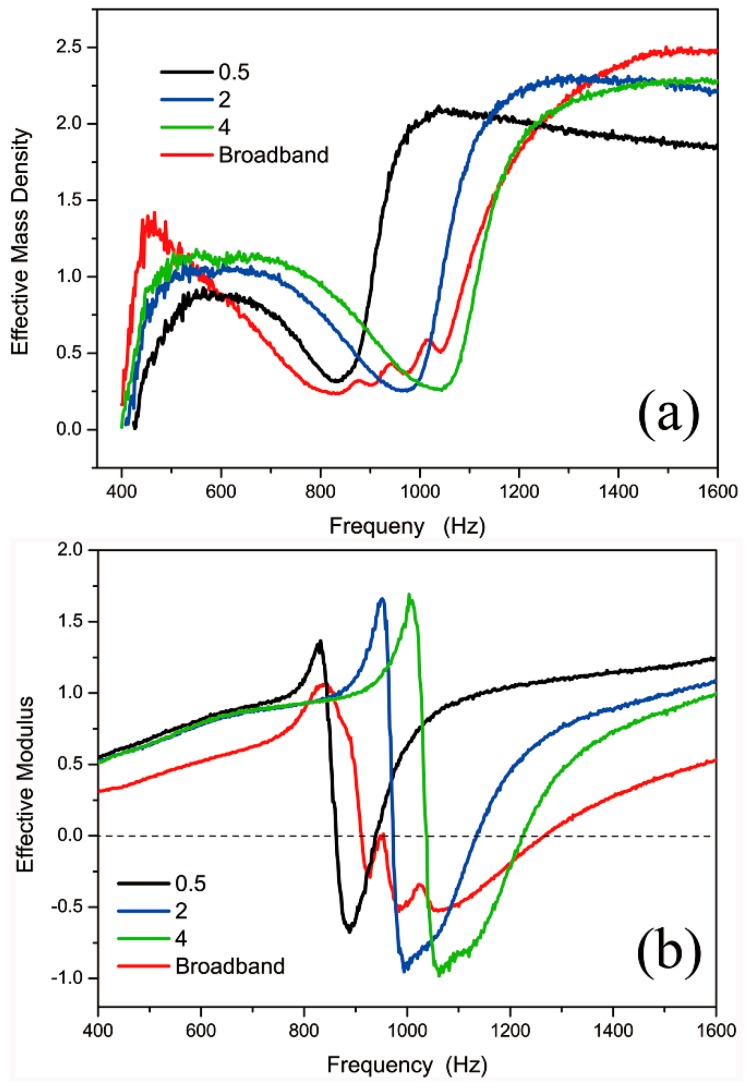
The effective parameters of broadband AMM: (**a**) effective mass density and (**b**) effective modulus.

To confirm the property of the broadband AMM, we retrieved the acoustic effective parameters of four kinds of AMMs. Figure 8 showed that the DSSHS AMMs with the distances of 0.5, 2, and 4 mm all had sharp changes in effective mass density and modulus near the resonant frequencies, respectively. The frequency range of the negative modulus was a little higher than that of the transmission dip. The mass density was always positive, but the modulus could reach negative values near the resonant frequency. As for the broadband AMM, the effective mass density was always positive, and the effective modulus was negative in a broadband frequency region of 900~1270 Hz, which was higher than the dip frequency range. It was possible that the different resonant units coupled with each other to present the negative modulus response in a higher frequency region. 

### 3.3. Transmitted Acoustic Metasurface with DSSHS

**Figure 9:**
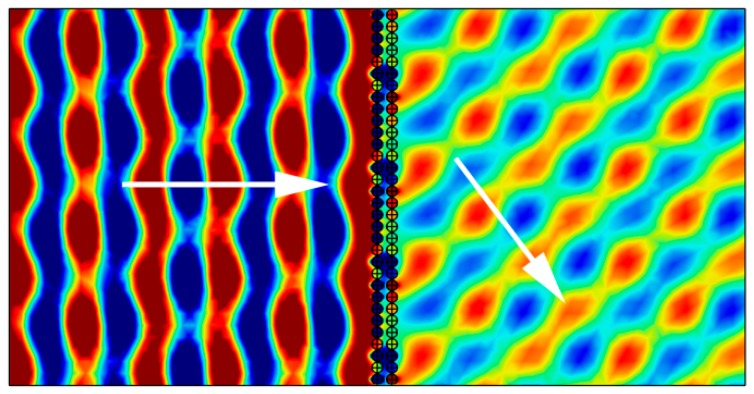
The transmitted acoustic field of the DSSHS metasurface. The white arrows indicate the direction of energy of acoustic waves.

Earlier research [22,23,24] illustrated that the SHS could only be used to design reflected AMS because the resonance of SHS was not strong enough. However, according to the analysis in Section 3.1, resonant coupling in the DSSHS structure could be utilized to improve the strength of the resonant response. With a stronger resonance, the DSSHS structure could produce a larger range of phase shifts for the transmitted waves. By selecting a suitable distance between each DSSHS in the structure, the transmitted phase shifts could cover 0~2π phase range. We designed eight kinds of DSSHSs with steps of π/5 phase shifts; These eight cells were spatially arrayed as a transmitarray in the matrix, as shown in Figure 9. The transmitted waves were calculated by the generalized Snell law (GSL).

(12) $$n_{2}\sin\theta_{t}-n_{1}\sin\theta_{i}=\frac{\lambda }{2\pi }\frac{d\Phi }{dx}$$

The transmitted acoustic field from the simulation is shown in Figure 9. The phase gradient of this kind of AMS was selected as dΦ/dx=π/64 rad/mm. When a 3640 Hz incident acoustic wave was perpendicular to the surface of the sample, the wave-front was manipulated according to the designed phase gradient. That is to say, the outgoing wave was obliquely refracted from the AMS. The refractive angle was 47° from the acoustic field distribution of the wave-front, which agreed well with the GSL formula of 46.9°. 

## 4. Conclusions

In conclusion, we proposed a resonant coupling method to achieve broadband AMM. Resonant coupling was caused by the mutual inductance of two resonators of SHSs, which generated abundant properties for incident acoustic waves. By tuning the distance of spit holes between two SHSs, we achieved different mutual inductances of the DSSHS, which resulted in different local resonant frequencies. The mutual inductances of the DSSHS can be used to design broadband AMM and transmitted AMS, which can achieve anomalous refraction of plane waves.
